# Knowing Our Enemy in the Antimicrobial Resistance Era: Dissecting the Molecular Basis of Bacterial Defense Systems

**DOI:** 10.3390/ijms25094929

**Published:** 2024-04-30

**Authors:** Mario Martínez, Irene Rizzuto, Rafael Molina

**Affiliations:** Department of Crystallography and Structural Biology, Instituto de Química-Física Blas Cabrera, Consejo Superior de Investigaciones Científicas, 28006 Madrid, Spain

**Keywords:** antimicrobial resistance, bacterial defense systems, phage therapy, CRISPR–Cas, abortive infection systems, bacterial defense islands

## Abstract

Bacteria and their phage adversaries are engaged in an ongoing arms race, resulting in the development of a broad antiphage arsenal and corresponding viral countermeasures. In recent years, the identification and utilization of CRISPR–Cas systems have driven a renewed interest in discovering and characterizing antiphage mechanisms, revealing a richer diversity than initially anticipated. Currently, these defense systems can be categorized based on the bacteria’s strategy associated with the infection cycle stage. Thus, bacterial defense systems can degrade the invading genetic material, trigger an abortive infection, or inhibit genome replication. Understanding the molecular mechanisms of processes related to bacterial immunity has significant implications for phage-based therapies and the development of new biotechnological tools. This review aims to comprehensively cover these processes, with a focus on the most recent discoveries.

## 1. Introduction

Antimicrobial resistance (AMR) stands as one of the most pressing global health challenges, jeopardizing the effective treatment of infections worldwide. In 2022, the inaugural comprehensive assessment of the global health impact of AMR revealed alarming figures, estimating that 4.95 million deaths in 2019 were linked to AMR, with 1.2 million directly attributable [1]. Despite confirming that AMR’s impact on morbidity, mortality, and disability rivals that of HIV and malaria, the incidence of AMR has escalated notably during the COVID-19 pandemic [2]. In addition, at the community level, another major concern to consider in microbial treatment is the formation of biofilms, especially if they are polymicrobial. Consequently, the growing AMR crisis and the relevance of bacterial biofilm formation have reinforced exploration into antimicrobial alternatives, with bacteriophage therapy emerging as a particularly promising avenue.

Phages, the natural predators of bacteria, use the host cell’s machinery to replicate. Upon specific binding to their host bacteria during adsorption, phages inject their genome into the host. Subsequently, phages exhibit two primary types of lifestyles. The lysogenic cycle involves integrating the phage genome into the host genome as a prophage, which is transmitted across bacterial generations. In contrast, the lytic cycle requires the expression of phage nucleic acids by the host cell machinery, resulting in the production of both the phage genome and proteins that assemble into new virions [3]. At the end of a lytic cycle, the host cell undergoes lysis, liberating the progeny phages. Each of these stages can be perceived by the defense systems of the bacterial cell as indicators of viral infection. Bacteriophages have co-evolved alongside bacteria for nearly 4 billion years. With an estimated number of 10^31^ bacteriophage particles inhabiting the biosphere, they are considered to be the most abundant biological entities on the planet [4], contributing to 20–40% of daily bacterial mortality [5] and impacting significantly on biogeochemical cycles [6]. The pressure of phage infection on bacteria has led to the evolution of sophisticated mechanisms through which phages manipulate and exploit their hosts, alongside a diverse array of bacterial immune mechanisms collectively referred to as antiphage defense systems [7]. These immunity systems encompass both innate mechanisms, such as restriction–modification systems, and adaptive mechanisms, such as CRISPR–Cas. While recent studies have started to unveil many previously uncharacterized defense systems, the full inventory likely extends well beyond current knowledge.

Research interests in bacterial–phage interactions, particularly bacterial defense, are multifaceted. Firstly, the identification of additional antiphage defense systems holds the promise of offering new insights into the ancient coevolutionary conflict between viruses and their hosts. Recent studies have revealed that many defense systems share homologs with similar functions in eukaryotic innate immunity, suggesting a potentially ancient origin and cross-kingdom conservation of these immune systems [8,9]. Secondly, previous investigations about antiphage defense have yielded precise molecular tools such as CRISPR and restriction enzymes. Therefore, the discovery of new immune mechanisms may lead to the development of novel tools for cellular and genomic manipulation. Thirdly, there is growing interest in utilizing phages to combat antibiotic-resistant bacterial infections and modulate microbiomes [10,11,12]. A deeper comprehension of the diverse mechanisms by which bacteria defend themselves may prove pivotal for these endeavors [13]. Overall, the significance of bacterial immune systems in these areas has generated renewed interest in the exploration and characterization of phage-resistance mechanisms. 

The bacterial antiphage arsenal has historically been considered to consist mainly of restriction–modification (RM) and abortive infection systems (Abi), with CRISPR–Cas added later [3,14]. However, advancements in high-throughput bioinformatic and experimental techniques have significantly broadened our understanding of bacterial antiphage mechanisms, revealing a wealth of more than a hundred diverse systems and mechanisms [15,16]. Consequently, while the prior knowledge of antiphage mechanisms was predominantly limited to systems detecting and degrading invading nucleic acids, recent discoveries have unveiled unexpectedly complex bacterial immune strategies. These include systems generating small antiphage molecules [17,18], systems relying on intracellular signal transduction through the production of signaling molecules [8,19,20] and systems recognizing conserved structural patterns of viral proteins to trigger immune responses [21]. These discoveries were facilitated by the tendency of defense systems to co-localize on bacterial and archaeal genomes, forming what are termed “defense islands” [22], although the precise reasons for their clustering remain unclear. Systematic analyses of defense islands in tens of thousands of microbial genomes, along with investigations into antiviral hotspots in phage genomes [23], have led to the discovery of several dozens of defense systems exhibiting a variety of defensive mechanisms. The genomic features of antiphage systems reflect their diversity. Firstly, the repertoire of genes associated with phage defense encompasses diverse functions and domains, including nucleases, helicases, proteases, kinases, ATPases, reverse transcriptases, and others. While the variety of this repertoire may seem relatively limited, comprising mostly nucleic acid-interacting domains and a few other domains (e.g., SIR2, TIR, and transmembrane (TM) domains), antiphage genes can be combined in unique ways to compose diverse defense systems. Some defense systems consist of single genes (e.g., AbiH, Lit, NixI, and BstA systems), while others may encode five genes or more (e.g., CRISPR–Cas, BREX, DISARM, Dnd, and Ssp systems). Furthermore, the abundances of different defense systems in bacteria vary greatly from one system to another. Thus, most genomes (78%) encode at least two known defense systems (with an average of six), and these are unevenly distributed across taxa, with some genomes containing dozens and others none [24,25,26]. The frequent association of defense systems with phages [27,28,29,30], phage satellites [23,31,32,33], integrative conjugative elements [34,35], and plasmids [36,37,38] suggests that genomic clustering may facilitate the horizontal dissemination of defense systems [24]. Additionally, defense islands may emerge due to synergy between defense systems and/or a need for coordinated regulation [39,40,41]. However, certain defense systems may exhibit antagonistic relationships that prevent their stable co-existence [42], but incompatibility can be overcome by mechanisms such as epigenetic silencing [43]. 

This review offers an encompassing view of the prokaryotic immune landscape, emphasizing recently discovered and characterized systems. Presently, these defense systems can be classified based on the immunity strategy adopted by bacteria, which correlates with the stage of the infection cycle [20]. Accordingly, bacterial defense mechanisms may involve degrading invading phage nucleic acids, inducing abortive infection or inhibiting phage DNA and RNA synthesis.

## 2. Degradation of Phage Nucleic Acids

The most prevalent defense systems in the microbial realm are those that focus on targeting and degrading phage nucleic acids.

### 2.1. Restriction–Modification Systems 

**Figure 1:**
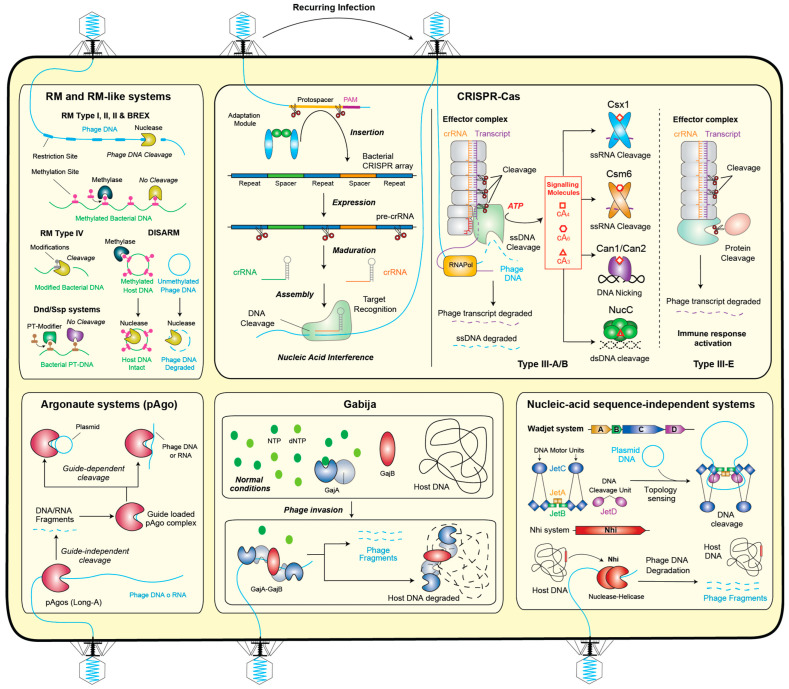
Antiphage bacterial defense systems based on the degradation of phage nucleic acids.

Restriction–modification systems serve to distinguish self from non-self, cleaving phage-invading nucleic acids based on epigenetic modifications [44]. These systems are widespread, occurring in over 95% of sequenced bacterial and archaeal genomes [45] and are extensively characterized owing to the biotechnological utility of restriction enzymes [46]. Typically, RM systems comprise at least two antagonist components: one recognizing specific sequences in the bacterial genome and modifying them, often by adenine or cytosine methylation, and another recognizing unmodified motifs in the DNA of the viral invader and cleaving it [46,47]. RM systems are categorized into four types (I–IV) based on their subunit composition and biochemical characteristics [45], with type IV restriction enzymes cleaving modified instead of unmodified DNA [47]. Some RM-like defense systems function differently: they may contain methyltransferases operating with other proteins such as proteases, phosphatases, and phospholipases, as seen in the phage growth limitation (Pgl) system [48,49]. Others distinguish self from non-self by the methylation of specific DNA sites, providing resistance to multiple unrelated phages upon first exposure, as exemplified by the BREX (bacteriophage exclusion) system [50,51]. There are also systems, like the defense island associated with the RM (DISARM) system, which use DNA topology instead of specific sequences to discriminate between self and non-self [52,53]. Additionally, other host DNA modifications can provide defense, such as those carried out by DndACDE and SspABCD protein complexes, which perform double-stranded and single-stranded phosphorothioate modification, respectively [54,55]. Similarly, the modification of the host chromosome with 7-deazapurine in DNA inhibits transformation with non-modified plasmids [56]. Overall, defenses based on epigenetic modifications represent the most prevalent and general immune mechanisms in prokaryotes [26] (Figure 1).

### 2.2. CRISPR–Cas Systems 

CRISPR–Cas systems represent one of the most prevalent defenses, being present in 42% of bacteria and 85% of archaea [57], and due to their programmability and precision, they have been developed into revolutionary genome engineering tools, therapeutics, and diagnostics [58,59]. These systems function by recognizing and interfering with phage nucleic acids through an adaptive immune response, enabled by their ability to generate and retain memories of past infections [60]. The immune memory is acquired through the adaptation complex, which incorporates short viral DNA sequences, termed “spacers”, into the bacterial genome. Subsequently, these sequences are transcribed, processed, and loaded onto the CRISPR–Cas interference machinery, where they guide it to specifically recognize viral DNA or RNA and prevent further infection. CRISPR–Cas systems are generally classified into two classes and six types based on the set of Cas genes they encode and the nature of their effector (single protein or protein complex) [61]. Type III CRISPR–Cas systems exhibit unique functions, degrading both DNA and RNA from invaders. Their response is initiated by the transcription of a target sequence by RNA polymerase (RNAP), which recruits the type III CRISPR–Cas effector complex Cas10-Csm/Cmr to the transcription bubble. Base pairing between the target transcript and the CRISPR guide RNA of the complex activates two distinct biochemical functions of Cas10: non-specific DNase activity on exposed single-stranded DNA by the HD domain and synthesis of cyclic oligoadenylate (cA) by the Palm domain. Both of these activities are inhibited by the sequence-specific cleavage of the target. The cyclic oligo-adenylate second messengers (cA_n_) activate CRISPR–Cas-associated proteins such as the Csm6/Csx1 family of RNases, Can1, which is involved in nicking supercoiled DNA, or NucC, which degrades dsDNA. However, as the unlimited action of cA_n_ molecules is toxic for the cells, prokaryotes have developed an ‘off-switch’ regulatory system based on cyclic oligoadenylate degrading enzymes termed ring nucleases [62]. In *S. islandicus*, for instance, a cyclic tetra-adenylate (cA_4_) is produced, which binds to Csx1 and activates its RNase activity, leading to the widespread degradation of RNA transcripts in the cell [63]. Then, to limit the degree of RNA degradation in the cell, two different types of ring nucleases are responsible for degrading cA_4_, thereby inhibiting the action of the Csx1 RNase [64,65]. Interestingly, this duplicity of ring nucleases is observed in other similar organisms as well. One explanation might be related to fine-tuning the immune response depending on different infection scenarios, but further studies are needed to clarify this point. This situation described above occurs in the well-characterized members from type III-A and type-III-B. However, recently, it has been discovered that atypical type III members, such as in type III-E, provide immune defense through distinct mechanisms [66] (Figure 1).

### 2.3. Prokaryotic Argonautes (pAgo)

Prokaryotic argonautes play a crucial role in the nucleic-acid-guided cleavage of phage DNA and RNA, aiming to inhibit viral propagation [67] (Figure 1). Argonaute proteins are ubiquitous across all three domains of life. Initially discovered in eukaryotes (eAgos) as components of interference for gene expression regulation, shorter pAgos contribute to defense against phages by collaborating with various known antiphage effectors [68,69,70]. pAgos can be classified into long pAgos and short pAgos. Long pAgos, whether they possess nucleic acid cleavage capabilities (long-A pAgos) or not (long-B pAgos), are involved in the nucleic acid degradation strategy. On the other hand, short pAgos lack the ability to cleave nucleic acids. Instead, they participate in immunity through a cell death mechanism known as abortive infection. It is noteworthy that due to their nucleic-acid-guided cleavage properties, long-A pAgos hold potential for programmable gene editing [71].

### 2.4. Gabija

The Gabija defense system comprises GajA and GajB components and is found in over 8.5% of prokaryotic genomes [15]. While GajB is predicted to be a UvrD-like helicase, GajA shares homology with overcoming lysogenization defect (OLD) nucleases [72,73]. Recent studies have revealed that GabjA and GabjB assemble into a large GajAB complex of approximately 500 kDa, comprising a tetrameric core of GajA subunits flanked by GajB dimers [74]. GajA acts as a site-specific endonuclease that nicks double-stranded DNA, presumably triggering an abortive infection response through the degradation of phage and host genomic DNA [72]. The activation of GajA occurs in response to changes in nucleotide concentrations in the cell, which may result from phage DNA replication and transcription [72] (Figure 1).

### 2.5. Nucleic Acid Sequence-Independent Systems 

Nucleic acid sequence-independent systems represent another type of bacterial defense mechanism. For instance, the antiplasmid Wadjet system was recently identified to recognize small invading plasmids through topology sensing and to cleave closed-circular DNA [75,76]. Thus, the sensing of characteristic structural features of newly injected phage DNA might also be utilized for antiphage defense, although supporting evidence for such a mechanism is currently lacking. Similarly, another sequence-independent defense mechanism has been elucidated for the nuclease-helicase immunity (Nhi) system, which relies on a multifunctional enzyme capable of detecting and degrading phage-specific DNA replication intermediates [77] (Figure 1).

## 3. Abortive Infection

Abortive infection systems constitute a significant strategy of bacterial defense against phages, functioning by inducing cell death upon the recognition of phage infection [78]. These systems terminate the cell before the maturation of the phage progeny, thereby halting the spread of phages to neighboring cells and safeguarding the bacterial community. Abi systems have been described since the 1950s, with various mechanisms characterized to date.

### 3.1. Signaling-Based Defenses

**Figure 2:**
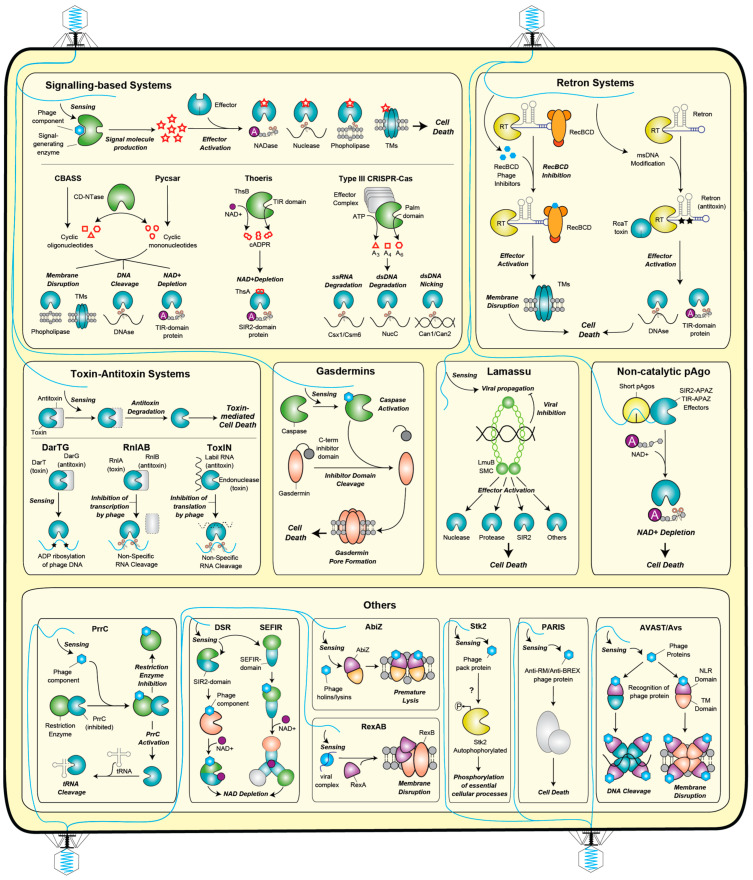
Antiphage bacterial defense systems based on abortive infection mechanisms.

Signaling-based defenses represent a common form of abortive infection systems wherein the recognition of phage infection triggers the production of a signaling molecule, subsequently activating a cell-killing effector [79,80]. These systems encompass diverse mechanisms, including cyclic-oligonucleotide-based antiphage signaling systems (CBASS), Pycsar, and Thoeris. *CBASS* systems share ancestry with the cyclic GMP-AMP synthase (cGAS) stimulator of interferon genes (STING) innate immune pathway in animals [8]. In CBASS systems, enzymes of the CD-NTase family produce cyclic oligonucleotides as the signaling molecule upon detecting phage infection, including specific structural components such as the phage capsid [81,82]. These signals activate an effector that promotes Abi through diverse mechanisms, including membrane impairment and DNA degradation [8,9,83,84,85]. CBASS systems exhibit diversity and are classified into types I–IV based on operon composition, signaling molecules, and effector functions [86]. They contain diverse effectors and oligonucleotide cyclase enzymes that synthesize various cyclic di- and trinucleotide activators such as cyclic cGAMP, cyclic UMP-AMP, cyclic UMP-UMP, and cyclic AMP-AMP-GMPs [87]. Similarly, the pyrimidine cyclase system for antiphage resistance (*Pycsar systems*) produces cyclic CMP (cCMP) or cyclic UMP (cUMP) upon phage infection, activating effectors to perform Abi through membrane impairment or depletion of cellular nicotinamide adenine dinucleotide (NAD+) [88]. In the *Thoeris defense system*, the recognition of the infection stimulates a Toll/interleukin-1 receptor (TIR) domain-containing protein (ThsB) to produce a signaling molecule that is an isomer of cyclic ADP-ribose (cADPR) [19]. This molecule binds and activates the toxic effector of the system, ThsA, that contains a sirtuin (SIR2) domain responsible for depleting the cell of NAD+, thereby triggering Abi [89]. Thoeris also resembles a eukaryotic immune pathway, as TIR domain receptors in plants produce a similar signal molecule upon pathogen recognition, leading to cell death via a mechanism reminiscent of Abi. Similarly, a situation akin to CBASS and Pycsar occurs in type III CRISPR–Cas, where the recognition of phage nucleic acids by the effector module triggers the production of a cOA signaling molecule (cOA) that arrests growth and ceases infection [90,91]. The presence of intermediate signaling molecules offers intriguing possibilities, allowing the cell to finely tune its response. For instance, ring nucleases associated with CRISPR type III degrade cyclic oligoadenylate molecules produced during infection, interrupting the Abi pathway before cell death if the infection is otherwise resolved [92]. Conversely, signaling systems offer the potential for signal amplification, as observed in the CRISPR type III system encoded by the archaea *Sulfolobus sulfataricus*, which produces thousands of signaling molecules per RNA molecule detected, enabling an early immune response [92]. Overall, the discovery of signal transduction through messenger molecules for antiphage defense reveals fascinating parallels with eukaryotic immunity (Figure 2).

### 3.2. Retron Systems

Retron systems are founded on the surveillance of the integrity of bacterial cell machinery. They consist of a reverse transcriptase (RT), a non-coding RNA (ncRNA), and accessory effector protein(s) [93]. The RT utilizes the ncRNA to generate a DNA-RNA hybrid (msDNA) that, in conjunction with a reverse transcriptase and effector proteins, safeguards cellular components [94]. Although the precise mechanism remains elusive, it is hypothesized that phage infection disrupts the retron complex, likely through interactions with the msDNA component, ultimately leading to cell death [82] (Figure 2).

### 3.3. Several Toxin–Antitoxin (TA) Systems 

Several toxin–antitoxin (TA) systems have been demonstrated to become activated upon phage infection, resulting in either cell death or growth arrest. These systems monitor the integrity of the host machinery by encoding a toxin component that targets essential cellular processes or components, along with a labile RNA or protein antitoxin that counteracts the toxicity [95,96]. Upon phage infection, the degradation of the labile antitoxin releases the toxin, leading to the death of the host cell (Figure 2).

### 3.4. Gasdermins 

Gasdermins constitute a potent antiviral strategy conserved among bacteria and animals. Bacterial gasdermins are proteins that contain a pore-forming domain homologous to those found in human cells, which remains stabilized in an inactive state with buried lipid modification. However, upon phage infection, they undergo activation by dedicated caspase-like proteases, which catalyze site-specific cleavage and the removal of an inhibitory C-terminal peptide. This release of autoinhibition triggers the assembly of large and heterogeneous pores, leading to the disruption of membrane integrity. This process mirrors an ancient form of regulated cell death shared between bacteria and animals, known as pyroptosis [97,98] (Figure 2).

### 3.5. Lamassu 

The Lamassu family of defense systems serves to safeguard prokaryotes against both viral infection and plasmid replication through abortive infection mechanisms [25,99]. These systems are identified in approximately 10% of all sequenced prokaryotic genomes and involve proteins belonging to the structural maintenance of the chromosomes (SMC) family [25]. Alongside the Lamassu-associated SMC protein (LmuB), these systems encode the LmuA protein, whose N-terminal domain can be substituted with various effectors responsible for executing Abi. Despite the precise mechanism of system activation remaining unknown, it is hypothesized that LmuB detects the DNA of the invading element by recognizing replication intermediates. This recognition event triggers ATP hydrolysis by the LmuB ATPase, leading to the activation of the Abi-inducing effector LmuA [99] (Figure 2).

### 3.6. Non-Catalytic pAgos

Non-catalytic pAgos, such as long-B pAgos and short pAgos, differ from catalytic pAgos (long-A pAgos) in that they lack the ability to cleave target nucleic acids due to containing a catalytically inactive PIWI domain [100]. However, these pAgos retain the capability for guide-mediated nucleic acid binding and function in conjunction with a variety of effector proteins that induce cell death upon target detection [68,101]. For instance, short pAgos form complexes with effectors, often consisting of fusions of different domains including analog-of-PAZ (APAZ) and either SIR2 or TIR domains. Short prokaryotic Agos associated with SIR2-APAZ or TIR-APAZ effectors constitute the SPARSA and SPARTA systems [101], respectively, and act as heterodimeric Ago–effector complexes. Additionally, short *Sulfolobus islandicus* (Si) Ago systems employ Aga1 with Aga2 effector proteins [70]. Upon phage infection, these systems acquire guide RNAs from the invader transcripts to facilitate the recognition of invading DNA, resulting in the catalytic activation of the effector domains. In SPARSA and SPARTA, SIR2 or TIR activation leads to NAD(P)+ depletion and subsequent cell death [69,101] (Figure 2). 

### 3.7. Other Abi Systems

Other Abi Systems include PrrC, a toxin that becomes activated when restriction enzymes are inhibited by phage proteins, and additional systems that, similar to Thoeris, decrease the level of NAD+-infected cells such as defense-associated sirtuin (DSR) [16] and SEFIR proteins [102]. Other Abi systems lead to cell death by membrane lysis such as in AbiZ, RexAB, or AVAST/Avs; by phosphorylating essential cellular processes (Stk2); by sensing phage bacterial immune system inhibition such as in RM or BREX systems; or by degrading host DNA (AVAST/Avs) (Figure 2).

## 4. Inhibition of DNA and RNA Synthesis

In recent years, several antiviral mechanisms have been discovered that directly target the processes of phage DNA and RNA synthesis, thereby hindering viral replication. 

### 4.1. Chemical Defense Systems

**Figure 3:**
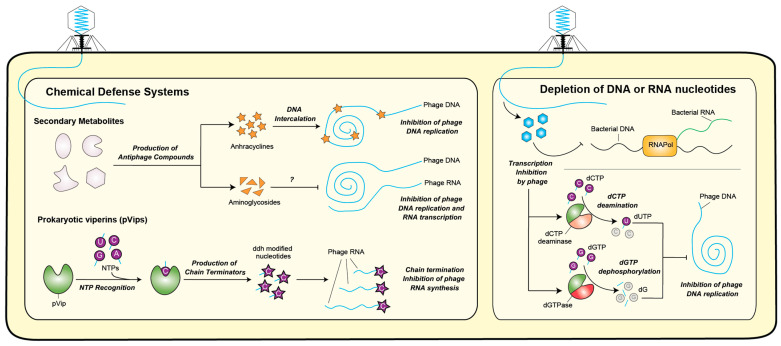
Antiphage bacterial defense systems based on the inhibition of DNA and RNA synthesis.

Chemical defense systems in bacteria produce small molecules that poison the synthesis of phage nucleic acids. *Anthracyclines*, for example, have been found to inhibit phage infection by potentially intercalating into phage DNA, thereby preventing DNA replication [18]. Since these secondary metabolites are secreted, they might provide antiviral protection to microbial communities acting in the early steps of invading phage DNA incorporation when it is still unpackaged [103]. Other secondary metabolites such as *aminoglycosidses* have also recently been shown to inhibit viral replication, potentially impacting transcription [17]. In an alternative mechanism, *Prokaryotic viperins* (pVips) were shown to produce several types of RNA chain terminator molecules [104], providing another mechanism for inhibiting viral replication (Figure 3). 

### 4.2. Depletion of DNA or RNA Nucleotides

Depletion of DNA or RNA nucleotides is an additional defense mechanism leading to DNA replication inhibition and is carried out by defensive enzymes that reduce deoxynucleotides, such as *dCTP deaminase* and *dGTPase* [88]. These are triggered upon infection to eliminate one of the DNA nucleotides, consequently affecting the ability of the phage to replicate its genome. In essence, these depletion mechanisms deprive the invaders of essential building blocks, ultimately halting their replication (Figure 3).

## 5. Conclusions and Future Directions

Over the past few years, numerous studies have uncovered a plethora of bacterial antiphage defense systems, many of which operate with their mechanisms still unclear. These systems have been lastly identified through their clustering with known defense mechanisms within ‘defense islands’, and subsequent experimental validation has confirmed their role in defending against phages [15,16,25]. However, not all defense systems can be easily detected using guilt-by-association approaches and necessitate strategies agnostic to genomic context [15,16,25]. Some systems may be rare or lack wide conservation, making it challenging to detect their enrichment within defense islands, and not all defense systems are necessarily linked to these islands. Additionally, the genomic-led discovery of new systems has been biased by pathogen-focused sequencing efforts resulting in significant gaps in our understanding of the broader role and importance of these defense systems in nature and across microbes. Hence, despite the significant advances made in mapping the bacterial defense repertoire, continued research efforts hold the promise of revealing novel defense mechanisms and providing fascinating insights [16]. 

There is still limited knowledge regarding the control of defense systems in bacteria. While for some systems, such as CRISPR–Cas, we are gaining a better understanding of their control mechanisms, indicating the necessity to regulate immune activation to manage defense-associated costs, the regulation of most defense systems remains completely unknown. Given that prokaryotes have multiple defense systems, some of which may interact [26], co-regulation is likely widespread, as observed for different CRISPR–Cas systems [105]. The induction of certain abortive infection systems is also the result of the coordinated activation of defenses, often interpreted as a last-resort protection when initial defense mechanisms fail. Interestingly, some Abi-inducing systems are proposed to adopt a “guarding” strategy, becoming activated only when an invading element inhibits other first-line defenses [23,106,107,108]. Insights into the triggers that activate defense systems upon infection are also being gained [21,109,110], but more work is required to identify other possible routes of regulation and activation. Remarkably, the prevalence of abortive infection systems, despite the significant fitness cost they impose on bacteria expressing them, underscores the importance of considering defense mechanisms not only at the single-cell level but also at the population level. Evidence of antiphage activity exhibited by certain molecules released by Actinobacteria further highlights the necessity of examining antiphage defense within microbial communities. Studies indicate that real-world factors like interspecies competition [111] and spatial heterogeneity may influence the dynamics of antiphage defense in bacteria [112]. However, the exploration of antiphage systems beyond controlled laboratory settings has been limited. Hence, the study of these systems under their natural environments could provide crucial insights into antiphage defense mechanisms.

The interplay between defense systems and the genetic elements encoding them appears to be far more intricate than previously thought. The diversity of defense systems arises not only from the ongoing conflict between bacteria and mobile genetic elements (MGEs) but also influences the fate of all parties involved. Bacteria, plasmids, phages, prophages, and phage satellites can all evolve and/or exploit existing defense systems, anti-defense systems, and even counter-anti-defense systems. These insights challenge the conventional view of defense systems as mere weapons in the battle between bacteria and phages. Instead, they suggest that antiphage defense systems can be seen as a versatile toolbox utilized by both MGEs and bacteria to influence gene flow, alongside notable antiplasmid and viral anti-defense systems [113]. Exploring a potential variety among antiphage defense, horizontal gene transfer (HGT) and/or mobile genetic element (MGE) regulation hold the promise of refining our current understanding of microbial genomics. This investigation could shed light on the interconnectedness of these processes and their collective impact on microbial evolution and adaptation. These breakthroughs prompt us to adopt a more integrated perspective on the role of defense systems within the intricate network of interactions between bacteria and phages. This necessitates moving beyond a simplistic view of how individual defense systems impact the dynamics of phage–host interactions.

Several hypotheses could explain the conservation of antiviral immune systems between prokaryotes and eukaryotes. One possibility is that these systems were inherited from the last common ancestor(s) of prokaryotes and eukaryotes [114]. Alternatively, some defense systems might have been acquired through horizontal gene transfer (HGT) events between prokaryotes and early eukaryotes. Combining the concepts of evolutionary tinkering [115] and immune building blocks opens fascinating research avenues. By delineating what can be considered antiphage building blocks, mapping them in chromosome genomes, and studying their possible combinations, researchers can systematically explore the diversity of antiphage systems in bacteria [116]. Moreover, the conservation of these building blocks across domains of life presents an opportunity to discover and understand eukaryotic immune mechanisms that share similarities with known antiphage systems. Likewise, the study of defense mechanisms in eukaryotes can contribute to the identification of defense systems in prokaryotes [104,117]. This is exemplified in cases such as in the conservation of both protein and domain structures between prokaryotic and eukaryotic immunity, as seen in Viperins [104], Gasdermins [97], TIR, and NLR domains [19,117]. This interdisciplinary approach could deepen our understanding of the evolution and adaptation of defense mechanisms against viral threats across the tree of life [89].

Collectively, the mechanistic richness of bacterial antiphage defense has led to significant biotechnological breakthroughs. Systems like RM, CRISPR–Cas, and retrons have yielded invaluable tools across various fields, including molecular biology, genome editing, and clinical diagnosis. Hence, it is anticipated that the continued exploration and characterization of defense systems hold great potential for future biotechnological and biomedical applications [47,118,119,120,121].

## Data Availability

No new data were created or analyzed in this study. Data sharing is not applicable to this article.
